# Estrogen Receptor Signaling in Radiotherapy: From Molecular Mechanisms to Clinical Studies

**DOI:** 10.3390/ijms19030713

**Published:** 2018-03-02

**Authors:** Chao Rong, Étienne Fasolt Richard Corvin Meinert, Jochen Hess

**Affiliations:** 1 Section Experimental and Translational Head and Neck Oncology, Department of Otolaryngology, Head and Neck Surgery, University Hospital Heidelberg, 69120 Heidelberg, Germany; fasolt.meinert@med.uni-heidelberg.de (É.F.R.C.M.); Jochen.Hess@med.uni-heidelberg.de (J.H.); 2 Research Group Molecular Mechanisms of Head and Neck Tumors, German Cancer Research Center (DKFZ), 69120 Heidelberg, Germany

**Keywords:** estrogen, estrogen receptor, radiotherapy, radioresistance, breast cancer, head and neck cancer

## Abstract

Numerous studies have established a proof of concept that abnormal expression and function of estrogen receptors (ER) are crucial processes in initiation and development of hormone-related cancers and also affect the efficacy of anti-cancer therapy. Radiotherapy has been applied as one of the most common and potent therapeutic strategies, which is synergistic with surgical excision, chemotherapy and targeted therapy for treating malignant tumors. However, the impact of ionizing radiation on ER expression and ER-related signaling in cancer tissue, as well as the interaction between endocrine and irradiation therapy remains largely elusive. This review will discuss recent findings on ER and ER-related signaling, which are relevant for cancer radiotherapy. In addition, we will summarize pre-clinical and clinical studies that evaluate the consequences of anti-estrogen and irradiation therapy in cancer, including emerging studies on head and neck cancer, which might improve the understanding and development of novel therapeutic strategies for estrogen-related cancers.

## 1. Introduction

**Figure 1:**
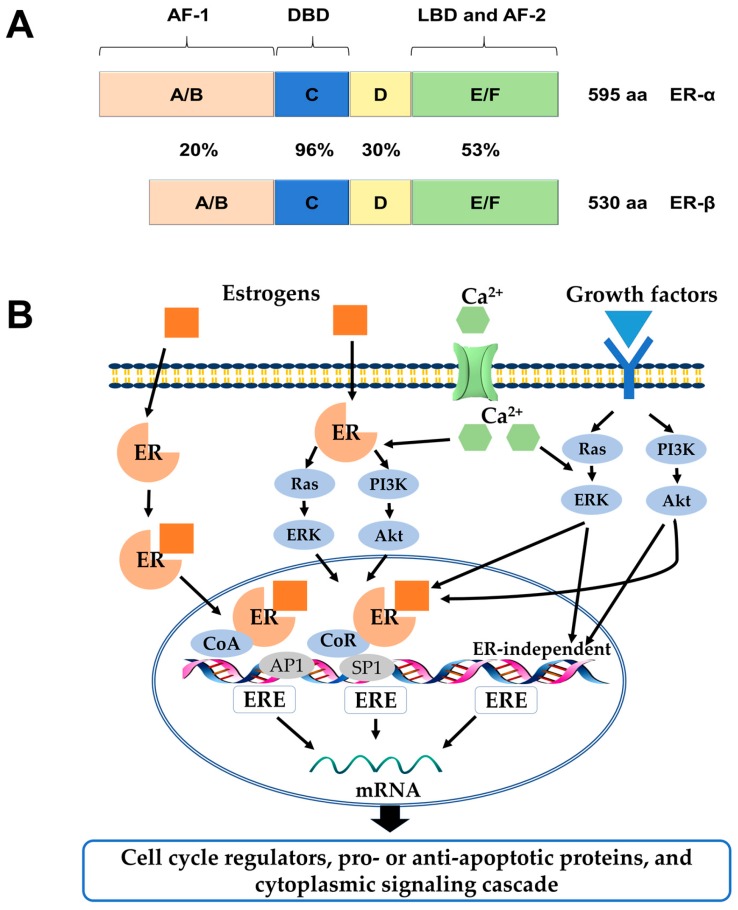
(**A**) Structural and functional domains of the ERα and ERβ. Structural domains of estrogen receptor α (ERα) (595aa) and ERβ (530aa) are labeled A-F. Both receptors have five distinct structural and functional domains: DNA-binding domain (DBD; C), hinge domain (D), ligand-binding domain (LBD; E/F), and two transcriptional activation function domains AF-1 (A/B) and AF-2 (F). The percentage of amino acid homologies between ERα and ERβ domains is also indicated; (**B**) Schematic illustration of ER-mediated signaling pathways. In the classical mechanism of ER action, estrogens (E2) bind to ERs and the E2-ER complex binds directly to estrogen response elements (EREs). Once bound to EREs the E2-ER complex can modify gene expression by the recruitment of distinct co-regulatory proteins, known as co-activators and co-repressors. In the ERE-independent genomic action, nuclear E2-ERs complexes interact with other transcription factors, such as activator protein 1 (AP1) or specificity protein 1 (SP1). In the ligand-independent genomic action, growth factors activate protein kinase cascades, such as Ras-ERK or PI3K-Akt, causing activation of nuclear transcription factors. In the non-genomic action, the E2-ERs complex activates protein-kinase cascades or cyclin adenosine monophosphate (cAMP) and calcium, leading to altered functions of proteins in the cytoplasm. ERK: extracellular signal–regulated kinase; PI3K: phosphatidylinositide 3-kinase; CoR: co-repressor; CoA: co-activator.

Estrogens exert many physiological functions in target tissues mainly via two members of the nuclear receptor superfamily: Estrogen receptor-α (ERα) and ERβ. They are encoded by separate genes, ESR1 and ESR2, respectively, transcribed from various chromosomal locations, and multiple mRNA splice variants exist for both receptors in normal and disease states [1,2]. On the structural level, both receptors possess five distinct structural and functional domains, harboring a DNA-binding domain (DBD), a ligand-binding domain (LBD), hinge domain and two transcriptional activation functions (AF-1, AF-2). As members of the hormone nuclear receptor superfamily, they share over 50% similarity in their hormone-binding domains and a predicted 96% similarity within the DBDs. However, there is a lower degree of sequence similarity within their hormone-independent AF-1 domains (Figure 1A) [3,4].

ERs and their variants mediate distinct effects as transcription factors in the nucleus when they are bound to their specific ligands through various mechanisms, which could be explained by genomic or non-genomic signaling pathways [5]. In the genomic mode of ER action, the ligands (e.g., estrogen hormones) diffuse into the cell and bind to the LBDs of the receptors, which result in homo- or heterodimer formation and subsequent binding to DNA at estrogen responsive element (ERE) sequences. Once bound to EREs the ligand-ER complex can modify gene expression by recruitment of distinct co-regulatory proteins, known as co-activators and co-repressors, or by interaction with other transcription factors, such as activator protein 1 (AP1), specificity protein 1 (SP1), and others [6]. In contrast, estrogen can elicit rapid response via non-genomic signaling pathways, which depend on the presence of a secondary messenger such as cyclic adenosine monophosphate (cAMP) and calcium, or the activation of protein kinases (Figure 1B) [5].

Over the last few decades, a growing number of studies have established a proof of concept that abnormal expression and regulation of ERs are crucial events in initiation and development of hormone-related cancers and are related to the outcome of cancer therapy. Many lines of evidence indicate that ERα and ERβ might perform different functions during carcinogenesis and anti-cancer therapy [2,7]. Currently, radiotherapy is used as one of the most common and potent cancer therapeutic strategies. It acts synergistically with surgical excision, chemotherapy and targeted therapy for treating malignant tumors in human. Encouragingly, clinical studies revealed that ER-positive breast cancer can be targeted by radiotherapy in combination with the modulation of ER activity, namely, endocrine therapy [8]. Tamoxifen belongs to the most frequently prescribed selective ER modulators (SERMs), which have been an effective and safe adjuvant endocrine therapy for several decades. However, the molecular mechanisms through which ionizing radiation (IR) regulates ER activity in cancer tissue and whether ER signaling has an impact on the efficacy of radiotherapy in various types of malignancies, remain largely elusive. Furthermore, the variability of ERα and ERβ expression, diverse response of ER and ER-related signaling to irradiation both contribute to the risk of safety and efficacy of cancer therapy.

In this review, we will discuss distinct functions of ER and ER-related signaling that are relevant to cancer radiotherapy. In addition, we will summarize pre-clinical and clinical studies that evaluate the consequences of anti-estrogen and irradiation therapy in cancer, including emerging studies on head and neck cancer, which might improve the understanding and development of novel therapeutic strategies for estrogen-related cancers.

## 2. Estrogen Receptor Signaling and Ionizing Radiation

### 2.1. Molecular and Cellular Responses to Ionizing Radiation

**Figure 2:**
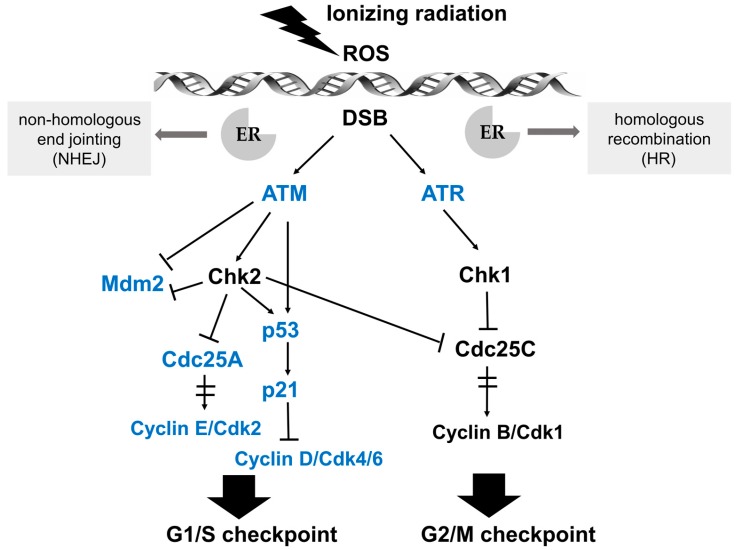
Schematic illustration of the signaling pathways in response to DNA damage and key effectors that interact with ER signaling. After perception of DNA lesions induced by IR directly or indirectly (by ROS generation), various biochemical signals are activated by cascades of protein kinases. Ataxia telangiectasia mutated (ATM) and ATM- and Rad3-related (ATR) kinases are upstream activators of IR-induced G1/S and G2/M checkpoint arrest. The G1/S checkpoint pathway is operated by p53 and Cdc25A in distinct branches. Firstly, ATM or Chk2 directly phosphorylates the p53 transcription factor and targets mouse double minute 2 homolog (Mdm2), achieving the stabilization and accumulation of the p53 protein. The critical effector of p53-dependent transcription is p21, which is a Cdk inhibitor and binds the complexes of Cyclin E/Cdk2 and Cyclin D/Cdk4/6. Another branch of the G1/S checkpoint pathway is activated rapidly via ATM-dependent phosphorylation of Chk2. Subsequently, Cdc25A, an activator of the Cyclin E/Cdk2 kinase, is degraded, preventing the activation of Cdk2. The ATM/Chk2-Cdc25A-Cdk2 axis accounts for the activation of the G1/S checkpoint via a p53-independent mechanism. In the G2/M checkpoint signaling pathway, the key downstream effector is the Cyclin B/Cdk1 protein complex, whose activation is restrained by ATM/Chk2 and ATR/Chk1 after IR-induced DNA damage. Moreover, Cdc25C phosphatase is also inhibited by Chk1/2 to activate the G2/M checkpoint. Key effectors that interact with ER signaling are marked in blue.

Radiotherapy is mainly based on the principle that normal tissue cells exhibit greater DNA repair capacity than carcinoma cells upon damage due to ionizing radiation [9]. Nowadays, ionizing radiation has become a widely applied treatment strategy for the majority of solid cancers. A series of biological effects on genomic DNA, which is considered as the most important target molecule, can be induced by photons, electrons, or heavy ions, which are generated by linear accelerators [10,11]. The biochemical lesions in genomic DNA of cancer cells can be achieved in a direct and indirect manner. A therapeutic dose of linear energy transfer (LET), such as particles or neutrons, can directly cause DNA damage, including single-strand breaks (SSB), modified bases, damage of the sugar backbone, double strand breaks (DSB) as well as effects on DNA repair [12,13]. Indirect effects are enforced by the generation of reactive oxygen species (ROS) that target and damage genomic DNA (Figure 2) [14]. It is worth noting that estrogens can also induce ROS in breast cancer cells, resulting in elevated genomic instability and a higher degree of clonal heterogeneity. The efficacy of radiotherapy might be modulated by estrogen-reduced ROS. The function of estrogen-induced ROS production in breast cancer has been reviewed previously by Okoh and coworkers [15]. Most types of human cells dispose of DNA damage with complicated response mechanisms, collectively named DNA-damage response (DDR), regardless of whether the damage is induced in a direct or indirect mode of action. Mechanisms of DDR can be activated and arrest the cell cycle at specific checkpoints, executing either DNA repair or induce programmed cell death (namely apoptosis) and cellular senescence, which are critical for maintaining cellular genomic integrity and for preventing neoplastic transformation [16].

DSB damage is the most lethal type of DNA damage induced by ionizing radiation [17]. DNA repair is the frontline response to cellular DNA damage, which also contributes to irradiation resistance in tumor cells. Efficient DNA repair enables tumor cells to replicate and survive. Generally, DSB damage repair is carried out by two major pathways: non-homologous end jointing (NHEJ) and conservative homologous recombination (HR), which have been extensively reviewed previously [18,19,20]. NHEJ is considered as the primary DSB repair pathway, which is activated throughout the cell cycle and relies on rejoining free DNA ends without the requirement for sequence homology. During this repair process, DNA strands of DSB sites are cut or modified, and the ligation of DNA ends are achieved directly and quickly regardless of homology, deletions or insertions. Although this makes NHEJ possibly error-prone, this mechanism can repair the DNA damage rapidly to eliminate potential genetic instability [21]. It is worth noting that estrogens have been shown to induce components of NHEJ in breast cancer cells and that therapeutic targeting of ERs result in irreparable DSB [22]. HR is widely known as a more precise mode of repair, which uses an undamaged template to retrieve the chromatid sequence content missing at the DSB sites. During HR, the damaged chromatid physically contacts with an undamaged sister chromatid with a homologous sequence for genetic information restoration in the late S/G2 phase of the cell cycle [23]. Compelling experimental evidence implicates that estrogens mediate both positive and negative regulation of HR. In melanoma, the tumor suppressor gene *MEN1* and ERα stimulate the transcription of *BRCA1*, *RAD51* and *RAD51AP1*, which encode key players in HR-directed DNA repair. Fulvestrant inhibits *BRCA1*, *RAD51* and *RAD51AP1* expression, resulting in decreased HR activity [24]. However, in medulloblastoma, an enhanced ERβ activity has been associated with nuclear translocation of insulin receptor substrate 1 (IRS-1), which interacts with *RAD51* at the sites of damaged DNA and reduces the HR function [25]. Pharmacological inhibition of ERβ induces medulloblastoma cells resistance to cisplatin by elevated formation of *RAD51* and increased levels of HR [26].

Cell cycle progression can be arrested at distinct cell cycle checkpoints temporarily, which are the G1 checkpoint during transition from G1 to S phase, and the G2 checkpoint of G2/M phase boundary. After perception of DNA lesions induced by IR, various biochemical signals are activated by well-defined cascades of protein kinases. Ataxia telangiectasia mutated (ATM) and ATM- and Rad3-related (ATR) kinases are upstream activators of IR-induced checkpoint arrest. ATM and ATR fulfill their physiological functions via phosphorylation of numerous substrates, such as Chk (checkpoint kinase) 2 and Chk 1, respectively, which are essential for cell cycle arrest at G1/S or G2/M in response to DNA damage [27,28]. The G1/S checkpoint pathway is mainly operated by two key effectors, namely the p53 transcription factor and the cell division cycle 25 A (Cdc25A) phosphatase, which regulate two distinct branches. The key effector for the G2/M checkpoint is the Cyclin B/Cdk1 protein complex, whose activation after IR-induced DNA damage is regulated by ATM/Chk2 and ATR/Chk1 (see Figure 2 for details) [29].

### 2.2. Interaction between Estrogen Receptor Signaling and Ionizing Irradiation

It is well established that estrogens regulate cell cycle progression in hormone-related carcinomas [30]. Therefore, the influence of estrogens or estrogen modulators on cell cycle progression is a critical factor for the interaction between ER signaling and IR. In MCF-7 cells, a major effect of estrogen is the activation of cell cycle progression by induction of G1 phase entry and shortening of the G1/S transition [31]. This effect is at least in part due to induced transcription of *c-Myc* and *Cyclin D1*, two key regulators of cell cycle progression [31]. Genomic approaches were applied to demonstrate that *c-Myc* regulates radioresistance through transcriptional activation of *Chk1* and *Chk2* by direct binding to their gene promoters in nasopharyngeal carcinoma cells, revealing a potential therapeutic strategy in reduction of radioresistance through blockade of the c-Myc-Chk1/Chk2 pathway [32]. In breast cancer, several studies provided a functional link between estrogen-related signaling and *c-Myc* regulated transcription [33]. Induction of *c-Myc* by estrogen is achieved via binding of ER to an atypical estrogen-responsive cis-acting element (ERE) in the promoter sequence [34]. Antisense c-Myc phosphorothioate oligonucleotides restrained proliferation of estrogen-stimulated cancer cells [35]. Moreover, induction of *c-Myc* in estrogen deprivation-arrested cells simulated the function of estrogen by restarting the cell cycle progression [36]. It is also worth noting that *Cyclin D1* has been involved in estrogen/anti-estrogen regulation of cell cycle progression by binding and activating *Cdk2* and *Cdk4*. Elevated mRNA levels of *Cyclin D1* precede modifications at the protein level, suggesting that the function of estrogen in Cyclin D1 protein expression is mediated at the transcript level [37,38]. Estrogen triggers transcription of *Cyclin D1* by a cAMP response element (CRE) in the promoter region [39,40]. Induction of Cyclin D1 leads to the formation of Cyclin E-Cdk2 complexes, which results in increased phosphorylation of pRb and S phase progression [41]. These complexes also contribute to decreased Cdk inhibitor p21^Cip1^ and p27^Kip1^ protein levels [31]. In the same study, estrogen-activated Cdk2 and DNA synthesis was restrained by antisense Cdc25A oligonucleotides, while inactive Cyclin E-Cdk2 complexes were reactivated by Cdc25A in vitro and in vivo, identifying Cdc25A as another grow-promoting effector of estrogen action [31].

Interestingly, a ligand-independent induction in ERα was observed in breast cancer cells after irradiation, which might be a consequence of the cell cycle arrest and related regulatory proteins [42]. Induced cell cycle arrest by low doses of X-ray could be abolished by 17β-estradiol, increasing survival of tumor cells and restraining cellular senescence by the regulation of p21 and Rb-related pathways, but independent of p53 [43]. Molinari and coworkers [44] found that estrogen treatment of breast cancer cell lines modified the intracellular distribution and functional activity of p53, indicating estradiol-induced inactivation of p53 might contribute to carcinogenesis of estrogen-dependent tumors. ERα has been reported to bind directly to p53 at target gene promoters, such as *CDKN1A* and *PCNA*, resulting in abrogation of p53 function. Moreover, 17β-estradiol promotes the interaction of ERα and p53, consistent with inhibition of p21 transcription [45]. Several studies have also shown that nuclear factor-κB (NFκB), a transcription factor regulating a variety of cellular processes, is linked to ER signaling in breast cancer. These studies suggest a critical role of a functional crosstalk between ER and NFκB in the resistance of cancer cells against endocrine and irradiation therapies [46,47].

**Figure 3:**
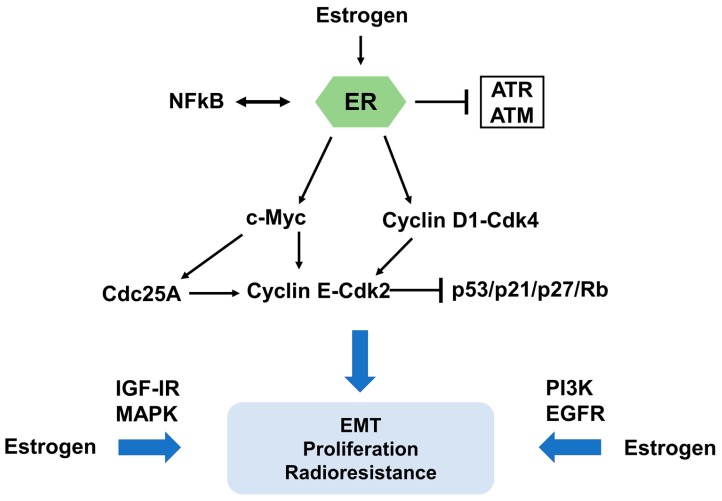
Molecular mechanism of estrogen and ER signaling contributions to radioresistance. The impact of estrogen and ER signaling on cell cycle progression is a critical factor for their contribution to radioresistance. c-Myc and Cyclin D1, two key regulators of cell cycle progression, have significant functions in estrogen and ER signaling mediated radioresistance. In addition, ER can interact with NFκB, a transcription factor, in resistance of cancer cells. Several protein kinase cascades, such as insulin-like growth factor I receptor (IGF-IR), mitogen-activated protein kinase (MAPK), phosphatidylinositol-3-kinase (PI3K), and epidermal growth factor receptor (EGFR) signaling, facilitate EMT, increased cell proliferation and enhanced radioresistance.

Besides transcriptional effects of estrogen, there are also non-genomic signaling pathways to be considered. Estrogen can induce growth factor signal cascades, including insulin-like growth factor I receptor (IGF-IR), mitogen-activated protein kinase (MAPK), phosphatidylinositol-3-kinase (PI3K), and epidermal growth factor receptor (EGFR) signaling, which trigger increased cell proliferation and enhanced radioresistance (Figure 1B) [48,49,50,51]. In ER-positive lung cancer, the EGFR directly phosphorylates ERα at specific serine residues [52]. Vice versa, estrogen triggers MAPK and PI3K/AKT signaling pathways to facilitate tumor metastasis through epithelial-to-mesenchymal transition (EMT) [53,54]. EMT is generally considered to be associated with radioresistance in distinct tumors [55,56,57,58]. Thus, estrogens exert a radioprotective function via genomic signal pathways and various classical growth factor pathways, indicating a rationale for anti-estrogen treatment to enhance the radiosensitivity of cancer cells (Figure 3).

## 3. The Combination of Anti-Estrogen and Irradiation Therapy in Cancer

Anti-estrogen therapy exerts functions by competing with estrogens for binding to ERs, most widely applied for the treatment of women with ER positive breast cancer. In 1971, a new anti-estrogen drug tamoxifen was reported firstly in the management of breast cancer [59]. Until now, tamoxifen reveals a significant clinical benefit and represents the most frequently prescribed anti-estrogen drug [59]. However, only a few studies exist that have addressed the potential value of tamoxifen therapy during or post radiotherapy.

Wazer and colleagues [60] investigated the interaction of tamoxifen and irradiation in the MCF-7 cell line. They observed that growth-inhibitory doses of tamoxifen reduced the radiosensitivity of breast cancer cells, indicating an enhanced repair of irradiation-related DNA damage. In order to unravel the effect of tamoxifen on radiosensitivity, Wazer and coworkers [61] extended the study on the ER-negative cell line MDA-MB-231, in which tamoxifen revealed no alterations in intrinsic radiosensitivity. They hypothesized that the interaction of estrogen, tamoxifen and irradiation in ER-positive breast cancer cells would be achieved by the regulation of the G1/S checkpoint. As introduced above, the G1/S checkpoint can be activated by irradiation to induce cell cycle arrest and to provide the time for DNA repair. This G1 phase block can be enhanced by tamoxifen and attenuated by estrogen. These observations have been confirmed by several studies with similar experimental conditions [62,63,64]. Interestingly, it has been demonstrated that anti-estrogen therapy can change radiosensitivity independent of the ER status, indicating that hormonal modulators might exert their effect via ERs but also in a non-receptor mode of action. Newton and colleagues [65] observed an enhanced apoptotic cell death in MCF-7 cells which were treated by a combination of irradiation and ZM182780, a pure anti-estrogen, or tamoxifen. In a more recent study, tamoxifen enhanced the radiosensitivity of human glioma cells by inducing cell apoptosis and sustaining G2/M arrest [66]. Moreover, fulvestrant, another pure anti-estrogen drug, revealed a positive effect on radiosensitization of ER-positive breast cancer cells by inducing cell cycle arrest and inhibiting proteins involved in DSB repair [67]. However, in contrast to these findings, Sarkaria and coworkers [68] reported that tamoxifen had no impact on radiosenstitivty of MCF-7 cells. The reason for these conflicting findings might be that MCF-7 cells exhibit an unusual form of apoptosis induction via activation of caspase-7 due to deficient caspase-3 activity [69]. Therefore, the MCF-7 cell line might not be an appropriate in vitro model to investigate irradiation or tamoxifen-induced apoptosis for breast cancer.

In order to further explore the interaction of anti-estrogen and irradiation therapy, in vivo studies have been conducted. Kantorowitz and colleagues [70] observed that a combination of tamoxifen and irradiation leads to a significant reduction of tumor volume and inhibits occurrence of additional tumors in a rat model of breast cancer induced by 1-methy-1-nitrosourea. Additional animal studies suggested that tamoxifen inhibits the initiation and promotion of irradiation-induced mammary tumors [71,72]. In line with these findings, anti-hormonal drugs, such as mifepristone, ICI182780 and Letrozole showed a sensitizing activity on chemo-radiotherapy in vitro and in vivo by increasing G2/M arrest in cervical cancer [73,74]. These data support the assumption that the interaction of anti-estrogen treatment and irradiation might be related to the regulation of cell cycle checkpoints. Although activation of cell cycle arrest by anti-estrogen drugs can facilitate DNA repair and eliminate irradiation-induced genomic lesions, its therapeutic activity might be due to the suppression of tumor cells repopulation after the irradiation interval. It is worth noting that concurrent treatment with anti-estrogen and irradiation therapy revealed an increased risk of lung fibrosis, cardiac damage and pneumonitis, which could be caused by the induced levels of transforming growth factor beta (TGF-β) [75,76,77,78].

In contrast to several in vitro studies, in vivo studies indicate a synergistic effect of concurrent tamoxifen and radiotherapy, which might be due to alterations in the tumor microenvironment. Several randomized controlled trials have shown that concurrent radiotherapy with tamoxifen achieved higher local control in breast cancer patients after lumpectomy compared to the treatment without tamoxifen, indicating that the combination of anti-estrogen and irradiation therapy is effective in the control of invasive cancer [79,80]. However, the optimal sequencing of endocrine therapy relative to radiotherapy remains elusive. An increasing number of retrospective clinical studies in breast cancer suggest that concurrent anti-estrogen and irradiation therapy shows no clear improved local control or favorable clinical outcome (Table 1) [81,82,83,84,85,86,87]. Moreover, the question of sequencing of hormonal and irradiation therapy for breast cancer has been addressed by several large randomized trials [87,88]. No significant difference between concurrent or sequential anti-estrogen therapy with irradiation was observed concerning clinical outcome of patients with breast cancer. Again, breast, lung or cardiac fibrosis was detected in patients with concurrent hormonal and irradiation therapy [89,90,91,92,93]. Therefore, it is reasonable for patients to receive hormonal and irradiation therapy sequentially to avoid the risk of toxicities. However, in view of the uncertainty and complexity of these trials, this conclusion should be treated with a great deal of caution. Furthermore, the complexity of the human immune response could explain the discrepancy between preclinical and clinical studies. Preclinical and clinical studies taking into account the paracrine interaction of tumor and immune cells, including novel immune-modulating therapies, are urgently needed to further address and to confirm this conclusion.

Although there is some evidence for a benefit of concurrent radiotherapy and anti-hormonal therapy in breast cancer, it might be more appropriate in other cancer entities. However, Dahhan and colleagues [94] reported a single case of low grade endometrial stromal sarcoma (ESS), where radiotherapy and anti-hormonal therapy were administered at the same time. This treatment resulted in tumor progression and was discontinued. Apart from that single case, no study has been published for other cancer entities that used concurrent radiotherapy and anti-hormonal therapy. Recently, a preclinical study conducted in our group provided experimental evidence for a causal link between ERβ expression and radioresistance in head and neck cancer [95].

## 4. ER Signaling in Head and Neck Cancer

In contrast to breast cancer, the role of estrogen and ER-related signaling is less well established in head and neck cancer (HNC). HNC is one of the most common human malignancies with around 600,000 new cases per year worldwide [96]. More than 90% of cases are diagnosed as head and neck squamous cell carcinoma (HNSCC), which develop from the mucosal epithelium of the upper aerodigestive tract. This includes oral cavity, nasopharynx, oropharynx, larynx and hypopharynx. Main risk factors are tobacco and alcohol consumption, human papilloma virus (HPV) and to a lesser extent Epstein–Barr virus (EBV) infection [97,98]. Current treatment options mainly consist of surgery, radiotherapy and chemotherapy, mostly platinum based [99].

Healthy tissues of human oral epithelium and salivary glands express mainly ERβ [100]. However, one study focusing on parotid gland pleomorphic adenoma found ERα and ERβ expression in normal tissue of the parotid gland especially in ductal cells. ERβ expression was enhanced in pleomorphic adenoma compared to normal tissue suggesting a possible role in tumor development [101].

There is also a possible role of ER signaling during HNSCC carcinogenesis. In a cell culture model, premalignant cells showed prominent ERβ but not ERα expression. Estradiol (E2) treatment induced Cytochrome P450 1B1 (CYP1B1), an enzyme that causes formation of carcinogenic metabolites from E2. E2 inhibited apoptosis but did not alter proliferation. In human tissue sections, ERβ showed distinct expression in normal tissue, dysplasia and squamous cell carcinoma (SCC) with most prominent staining in SCC [102]. In contrast, ERα expression was almost absent, suggesting a more prominent role of ERβ-related signaling. On the contrary, when using an artificial overexpression system for ERα combining with E2-treatment, Sumida et al reported an increase in proliferation and expression of EMT-markers [103].

First in vivo evidence for a causal role of ER-related signaling in the pathogenesis of HNSCC emerged in the late 1980s. In a mouse xenograft model for laryngeal cancer estradiol treatment enhanced the kinetic of tumor formation and tumor size [104]. Although there are some studies suggesting that HNSCCs express mostly ERα rather than ERβ [105], most research points in the opposite direction, claiming ERβ outweighing ERα. Two studies reported a more favorable outcome of ERβ positive tumors as compared to ERβ negative tumors [95,106]. Tumors positive for ERα, the effects of which are reported to be counteracted by ERβ, have been associated with slightly poorer survival [102,107]. However, it is worth noting that those results were produced investigating HNSCC from different primary sites and were based on different detection methods for ER expression.

It has also been shown that estrogen signaling exerts different biological effects in HNSCC tumor cells. E2-stimulation activates MAPK signaling in an additive fashion with EGF and induce invasion of HNSCC tumor cells [48]. This activity could be mediated by ERs or GPER1 (G protein-coupled estrogen receptor 1), which was shown to trigger proliferation and migration in laryngeal squamous cell carcinoma (LaSCC) via Interleukin-6(IL-6) and signal transducer and activator of transcription 3 (STAT3) [108]. There are also experimental data indicating that ERβ causes an increase of NOTCH1 expression and thereby favors differentiation in SCCs, including HNSCC. In line with this assumption, ERβ overexpression or treatment with specific agonists inhibited proliferation of SCC cell lines, including HNSCC [109].

More recently, our laboratory demonstrated an accumulation of ERβ-positive HNSCC cells after fractionated irradiation in vitro, suggesting a critical role of ERβ-related functions in radioresistance. Indeed, tamoxifen or Fulvestrant treatment revealed a sensitization of these cells to irradiation, which was accompanied by augmented apoptosis. Radioresistant tumor cells were also positive for submaxillary gland androgen-regulated protein 3A (SMR3A), which is a putative ERβ downstream target and was shown to serve as a prognostic biomarker for HNSCC patients. Accordingly, HNSCC with a high ERβ and SMR3A expression pattern were significantly associated with an unfavorable progression-free survival and disease specific survival [95].

## 5. Conclusions and Perspectives

The controversial findings of in vitro and in vivo preclinical studies in breast cancer indicate an enormous complexity and context-dependency with a strong impact on the efficacy of anti-estrogen treatment in combination with radiotherapy. Many questions are waiting to be resolved including better understandings of estrogen and estrogen modulators action on normal and cancer cells, precise mechanisms of interaction of estrogen signaling and irradiation, the development of novel estrogen modulators, as well as effective therapeutic strategies of combination of endocrine therapy and radiotherapy. Moreover, the distinct expression of ER subtypes in various cancer tissues, components of the ER-related signaling cascade, and regulation of many transcription factors, all contribute to a complex situation that impedes the therapeutic efficiency of endocrine therapy and radiotherapy. Therefore, not only breakthroughs from basic and preclinical studies but also translational clinical trials are urgently required to further explore and develop the combination therapies in distinct malignancies. Finally, the oncology research community including academic, hospital, industry and government will need to overcome challenges and achieve an encouraging therapeutic outcome for cancer patients.

## Figures and Tables

**Table 1:** Summary of clinical studies comparing concurrent and sequential anti-estrogen and irradiation therapy in breast cancer.

Type	Treatment Groups (*n*)	Tamoxifen or Aromatase Inhibitors	Radiotherapy	Chemotherapy (*n*)	Follow-up	Outcome	Reference
Retrospective 1976–1999	Concurrent (254) vs. Sequential (241)	generally for 5 years	48 Gy in 2 Gy Fractions with boost to primary tumor bed median total dose 64 Gy	CMF based (71) Adriamycin (42) other (16) none (371)	10.4 years	No difference in overall survival (OS), HR, 1.234; 95% CI, 0.42 to 2.05; No difference in local recurrence, HR, 0.932; 95% CI 0.42 to 2.05	[81]
Retrospective 1980–1995	Concurrent (174) vs. Sequential (104)	20 mg OD or 10 mg BID	Tangents only (182) or tangents and nodal (95) median total dose 64 Gy	Methotrexate-based (67) Doxorubicin-based (44) None (167)	8.6 years	No difference in OS, HR 1.56; 95% CI, 0.87 to 2.79; No difference in relapse-free survival, HR 1.23; 95% CI, 0.63 to 2.41; No difference in local recurrence, HR 1.22; 95% CI, 0.33 to 4.49; No difference in cosmesis, or significant complications.	[82]
Retrospective 1989–1993	Concurrent (202) vs. Sequential (107)	20 mg daily for 5 years	45–50 Gy to whole breast	cyclophosphamide, methotrexate, and fluorouracil (CMF) (156) cyclophosphamide, doxorubicin, and fluorouracil (CAF) (153)	10.3 years	No difference in OS, HR 0.84; 95% CI 0.40 to 1.78; No difference in local recurrence, HR 0.73; 95% CI, 0.26 to 2.04; No difference in grade 3 or 4 hematologic toxicity.	[83]
Retrospective 2001–2008	Concurrent (113) vs. Sequential (151)	anastrozole 1 mg or letrozole 2.5 mg daily for 5 years	50 Gy in 2 Gy Fractions with boost to primary tumor bed median total dose 63.2 Gy	CMF (1) Taxane-based (7) Anthracycline-based (31) Combination of anthracycline and taxane (6)	2.9 years	No differences in clinical outcome and treatment-related complications	[84]
Retrospective 2001–2009	Concurrent (158) vs. Sequential (157)	anastrozole 1 mg or letrozole 2.5 mg daily for 5 years	50 Gy in 2 Gy fractions with a boost of up to 63.2 Gy	Yes (57) None (258)	5.6 years	No difference in disease-free survival. No difference in Grade 3 or 5 toxicities	[85]
Retrospective 1998–2008	Concurrent (57) vs. Sequential (126)	Anastrozole or Tamoxifen	45–54 Gy over an average of 49.5 days	anthracycline or taxane (51) none (132)	2.3 years (Con) 2.6 years (Seq)	No difference in detectable breast fibrosis Concurrent (1.8%) vs. Sequential (4%) in Local recurrence	[86]
Randomized 2005–2007	Concurrent (75) vs. Sequential (75)	2.5 mg Letrozole daily for 5 years	A total dose of 50 Gy in 2 Gy fractions	FEC (28) None (122)	2.2 years	No difference in subcutaneous fibrosis, lung fibrosis and quality of life	[87]
